# The origins of microbial adaptations: how introgressive descent, egalitarian evolutionary transitions and expanded kin selection shape the network of life

**DOI:** 10.3389/fmicb.2014.00083

**Published:** 2014-03-04

**Authors:** Eric Bapteste

**Affiliations:** ^1^UPMC, Institut de Biologie Paris Seine, UMR7138 ‘Evolution Paris Seine’Paris, France; ^2^CNRS, Institut de Biologie Paris Seine, UMR7138 ‘Evolution Paris Seine’Paris, France

**Keywords:** evolution, tree of life, greenbeard genes, black queen theory, lateral gene transfer, symbiosis, mobile genetic element

## Microbial evolution: a genealogical perspective

Protists, bacteria and archaea (the prokaryotes), and their mobile genetic elements populate the microbial world. This world is ancient (several billion years old), numerically huge (with 5 × 10^30^ prokaryotic cells and 10–100 times more viruses!), and genetically extremely diversified. Such a large assemblage cannot be ignored in attempts to understand life's history on Earth, however, how can biologists account for its evolution?

For long, the notion of descent with modification, describing a process of vertical inheritance, defining a tree-like genealogical pattern, when the genetic material, modified by some mutations, is transferred from the genome of a last common ancestor to its direct progeny has offered a promising way to classify organisms and species. Thus, the origin of microbial adaptations can be searched for within lineages, in the changes of genetic material inherited from one ancestor. Yet, such studies are strongly constrained. Non-tree like evolution, generating a reticulate evolutionary pattern, cannot be analyzed with a genealogical tree. Moreover, viruses do not all originate from a single last ancestor (Lima-Mendez et al., 2008), nor do they all display obvious genealogical relationships with cellular organisms, hindering the collective study of mobile genetic elements and cells with a single tree.

Genealogy also plays a central role for explaining the main types of behaviors described in the biological world: selfishness, mutualism, altruism, and spite (West et al., 2006). The evolution of these interactions can be understood by accounting for kinship between protagonists, under the standard assumption that genealogical proximity between individuals entails their genetic proximity (Huneman, 2013). Thus, knowing the relative kinship, the benefit for the recipient of an interaction and the cost for its actor allows determining when an individual cooperating with a kin, in ways enhancing its reproduction, or preventing distantly related members of a population to reproduce, actually maximizes the reproductive success and the survival of its own genes (van Baalen, 2013). Microbiologists are thus inclined to embrace the conceptual framework of kin selection to analyze many cooperations (Diggle et al., 2007).

However, it is probably not enough to take genealogical relationships into account to explain the diversity, evolution, and interactions in the microbial world. Too systematic a focus on genealogy may even introduce some biases in the explanations of microbial diversity, evolution and interactions, because many crucial biological phenomena result from processes orthogonal to vertical descent.

## Expanding the analytical framework inspired by genealogy

The multiplicity of evolutionary processes, their consequences, and the interactions at play within the microbial world are still under-appreciated. The genealogical perspective grounding evolutionary explanations needs to be completed, because its analytical framework does not accommodate for numerous important biological phenomena, which deeply challenge our background knowledge of the (microbial) world and its evolution.

### Introgression: a class of non-tree-like evolutionary processes

Due to introgressive descent, many adaptations originate from outside rather than from within lineages of vertical descent. While in vertical descent, the genetic material of a particular evolutionary unit is propagated by replication inside its own lineage, in introgressive descent, the genetic material of a particular evolutionary unit propagates into different host structures and is replicated within these host structures (Bapteste et al., 2012). Such host structures are genealogically composite, made of components with distinct genealogical origins. Importantly, introgression is very common in the microbial world, affecting entities from the same or different levels of biological organization. New introgressive mechanisms are constantly discovered (Bapteste, 2013). However, these mechanisms and their actors (viruses, plasmids, conjugative elements, outer membrane vesicles, gene transfer agents, nanotubes, membrane fusion, …) are largely missing from the traditional evolutionary representation. For instance, a gene sequence can propagate into another gene sequence, creating a novel composite gene, whose components come from two different gene lineages. Similarly, a gene sequence can propagate within a genome, whose ancestor lacked this gene, producing a composite genome with genes originating from different genomes. Likewise, the genome of a mobile element (a virus, a plasmid, etc.) can propagate into a cell born without this element, creating a composite cell with genetic instruction from multiple sources. Or, a part or an entire microbial genome can propagate within a symbiotic association, producing a holobiont with several unrelated genomes. Therefore, the recognition of introgression promotes a substantial expansion of the evolutionary research program: a study of the origins (rather than of the origin) of adaptations and species through the description and analysis of a plurality of processes and objects, some unexpected in the traditional genealogical perspective (Doolittle and Bapteste, 2007; Bapteste, 2013).

### Gene–gene introgression: massive gene remodeling

Homology guides comparative analysis in evolutionary biology (Haggerty et al., 2013). Sequences or organs are considered homologous when they evolved in a tree-like fashion from an ancestral form. Thus, gene evolution is often described by a tree with one genealogy per gene family. However, many genes originate from the composition of genetic material from sequences belonging to different gene families. Eukaryotes are the main creators of composite genes (in terms of the proportion of composite genes in their genomes) (Haggerty et al., 2013), yet in terms of absolute numbers, mobile genetic elements operate the most massive gene remodeling on Earth (Jachiet et al., 2013). Therefore, numerous genes display family resemblances (Halary et al., 2013): true similarity caused by introgression between non-homologous sequences. Such family resemblances support the study of the origins of genes and of adaptations at a more global scale than delineated by homology.

### Gene–genome introgression: remarkable pangenomes

All conspecific individuals do not own the same gene families. Six percent only of their gene families are distributed in all 60 strains of *Escherichia coli* (Lukjancenko et al., 2010), and experiments showed that only 61 genes out of 246,065 cannot be transferred into an *E. coli* (Sorek et al., 2007). Members of this species (and many others) exploit a large DNA pool, called pangenome, larger than the size of individual genomes. Therefore, sequencing one individual genome does not always allow describing genetic and functional diversity at the species level. Pangenomes and lateral gene transfer –by small segments or larger chunks- are not restricted to conspecifics (Nelson-Sathi et al., 2012). These observations challenge phylogenetic systematics: they mean that genome evolution is much more than genome genealogy, an increasingly elusive concept, since these objects prove to be ever more composite and their genes do not all coalesce in a single common ancestral genome.

### From the mobilome network to the social network of life

Many classes of evolutionary objects (i.e., virus, plasmids, etc.) have fuzzy borders, because many of these objects do not evolve independently at the genetic level. Remarkably, introgression creates novel introgressive mechanisms. Numerous genealogically mosaic mobile elements (autonomous or not: polintons, virophages, R391, phasmides, phage inducible chromosomal islands, transpovirons, etc.) emerge and evolve through the sharing of mobility functions, defining a genetic pool: the pangenome of mobile elements, which unravels a network of shared genes between these elements (Yutin et al., 2013). This network belongs to a larger one: the social network of life, whose edges describe an important biological structure: “what shares genetic material with what,” without prejudices about the process involved in these sharings (in part vertical descent, but also introgression since these sequences can be used as common goods by more than one lineage Halary et al., 2010; McInerney et al., 2011). In this latter network, all entities are not genealogically related, but this does not imply their a priori exclusion from the model. Thanks to its diversity of edges and nodes, the social network of life is more inclusive than the tree of life, supposed to be universal but in fact restricted to one type of relationships between one fraction of biological diversity (Halary et al., 2010).

### The challenging microbial social life

Microbial social life is hard to explain within the framework of kin selection without (at least) deeply expanding this theory. How do bacteria manage to identify their kins and cooperate? In principle, greenbeard genes provide a way to detect other organisms carrying these genes with which an individual can act cooperatively. However, experimental transfers between strains and species of myxobacteria (*M. xanthus* and *M. fulvus*) of the first characterized single greenbeard prokaryotic gene predictably transform their interactions, reprogramming their social interactions. For example, when an isogenic *M. xanthus* strain expresses a *M. fulvus traA* allele, both become efficacious partners. Moreover, strains constructed with two alleles of *traA* cooperate with a broadened range of partners (Pathak et al., 2013). Consequently, the notion of microbial greenbeard gene departs from classical kinship selection: the cooperative behavior targets other individual harboring the same allele, whatever their global genetic proximity. Lateral gene transfer does not only partly uncouple gene and genome evolution, which makes it difficult to conceive of a standard application of kinship within bacterial populations, since bacteria may be similar for some genes without being similar for all, but the transfer of greenbeard genes can also induce cooperation between relatively different microbes. Therefore, cooperation between distantly related individuals must be more largely theorized (Huneman, 2013). The black queen theory provides a good instance of such an explanation in which genealogical relationships between protagonists do not play a role (Morris et al., 2012; Sachs and Hollowell, 2012). This theory would explain why a minority of organisms (*Synechococcus* harboring the *katG* gene) are sufficient to reduce the HOOH in ocean surface waters to a level that allows the dominant types (*Prochlorococcus* and *Candidatus Pelagibacter ubique* who lost this gene) to thrive.

Considerations on the evolution of social life are fundamental: our inability to grow the vast majority of micro-organisms in pure cultures (Staley and Konopka, 1985) may largely come from our too limited knowledge on this topic. Furthermore, our general knowledge in evolutionary microbiology mostly rests on analyses of the rare microbes able to grow in pure cultures. If these organisms are not representative of most of the microbial world, inferences based on a part of this world (e.g., the cultivable microbes) could be mistakenly conflated with general conclusions, which one hopes to be relevant for the whole microbial world. Yet, discoveries such as the Pandoraviruses remind us that much unknown lives outside our Petri dishes (Philippe et al., 2013). Importantly, alleviating some constraints inspired by the genealogical focus is one way to better see the whole rather the parts. Typically, sequences comparison free from the constraints of multiple alignment and a tree-based representation of sequence similarities hints at highly divergent environmental gene forms and lineages, not yet reported in the microbial world (Lynch et al., 2012).

### Egalitarian evolutionary transitions and systems with microbial components

The three steps of evolutionary transitions: the association of entities, their stabilization, and their transformation (after which entities originally able to reproduce independently are only able to reproduce as part of a larger whole) result either in fraternal transitions (which can be explained by traditional kin selection), when higher level units emerge from genealogically like components, and in egalitarian transitions, when higher level units emerge from genealogically different components (Huneman, 2013). These latter transitions are common in the microbiological literature, e.g., *Parakaryon myojinensis* (Yamaguchi et al., 2012), the origins of eukaryotes (Alvarez-Ponce et al., 2013), mutualistic viruses and even virophages, seen as components of larger systems (Espagne et al., 2004; Fischer and Suttle, 2011; Roossinck, 2011). The notion of egalitarian evolutionary transition gives credits to the proposal that some elements of microbiomes deserve to be considered as new organs, providing novel physiologies, despite their genealogically different origin from that of the host of these organs (Stahl and Davidson, 2006). The idea that the microbial component influences the physiology and the behavior of the other components of such systems is becoming increasingly popular (Hoover et al., 2011; McFall-Ngai et al., 2013), i.e., the study of mechanisms of the microbial-brain axis, testifying of the fundamental relevance in terms of adaptations of the interactions between the microbial and macrobial worlds (Collins et al., 2012).

Consequently, many evolutionary objects previously studied as if they were genealogically cohesive organisms or species would rather constitute genealogically heterogeneous systems, composed in parts by microbes or viruses. This microbial contribution to microbe–microbe and microbe–macrobe systems seems a general rule rather than an exception, when one considers the age, abundance, and ubiquity of these minute entities on the planet. This type of discoveries raises a novel fundamental issue: how to model the evolution of systems (and their possible physiological, ecological, and developmental impacts during Earth history), which brings microbial evolutionists very far from the usual reconstruction of a genealogical tree.

## Conclusion

Genealogical tree-thinking should at least be completed by other perspectives (Doolittle and Bapteste, 2007; Bapteste et al., 2012, 2013). For example networks can be used to adapt current models to the data rather than enforcing the data to fit within pre-existing genealogically constrained models, designed in order to analyze objects and processes far less complex than those affecting the microbial world (Bapteste, 2013).
